# Ether Spray as a Cure for Neuralgia

**Published:** 1883-03

**Authors:** 


*Ether Spray as a Cure for Neuralgia.*—Dr. McColganan in the *Southern Practitioner* extols the value of the ether or rhigolene spray for the instantaneous relief principally of facial neuralgia. Since curing himself, he has had occasion to test its efficacy in about twenty cases. The result was invariably a most gratifying success. In many instances a permanent cure was established. He attempts to explain its action by supposing a complete change to take place in the nutrition of the affected nerve in consequence of the intense cold acting as a revulsive.

